# Associations between Maternal Diet, Body Composition and Gut Microbial Ecology in Pregnancy

**DOI:** 10.3390/nu13093295

**Published:** 2021-09-21

**Authors:** Meghan L. Ruebel, Stephanie P. Gilley, Clark R. Sims, Ying Zhong, Donald Turner, Sree V. Chintapalli, Brian D. Piccolo, Aline Andres, Kartik Shankar

**Affiliations:** 1 Department of Pediatrics, Section of Nutrition, Anschutz Medical Campus, School of Medicine, University of Colorado, Aurora, CO 80045, USA; meghan.ruebel@cuanschutz.edu (M.L.R.); stephanie.gilley@cuanschutz.edu (S.P.G.); 2 Arkansas Children’s Nutrition Center, Little Rock, AR 72202, USA; crsims@uams.edu (C.R.S.); zhongying@uams.edu (Y.Z.); dturner@uams.edu (D.T.); svchintapalli@uams.edu (S.V.C.); BDPiccolo@uams.edu (B.D.P.); AndresAline@uams.edu (A.A.); 3 Department of Pediatrics, Section of Developmental Nutrition, University of Arkansas for Medical Sciences, Little Rock, AR 72205, USA

**Keywords:** diet, microbiome, obesity, pregnancy, adiposity, metabolic syndrome

## Abstract

Maternal body composition, gestational weight gain (GWG) and diet quality influence offspring obesity risk. While the gut microbiome is thought to play a crucial role, it is understudied in pregnancy. Using a longitudinal pregnancy cohort, maternal anthropometrics, body composition, fecal microbiome and dietary intake were assessed at 12, 24 and 36 weeks of gestation. Fecal samples (*n* = 101, 98 and 107, at each trimester, respectively) were utilized for microbiome analysis via 16S rRNA amplicon sequencing. Data analysis included alpha- and beta-diversity measures and assessment of compositional changes using *MaAsLin2*. Correlation analyses of serum metabolic and anthropometric markers were performed against bacterial abundance and predicted functional pathways. α-diversity was unaltered by pregnancy stage or maternal obesity status. Actinobacteria, *Lachnospiraceae*, *Akkermansia*, *Bifidobacterium*, *Streptococcus* and *Anaerotuncus* abundances were associated with gestation stage. Maternal obesity status was associated with increased abundance *of Lachnospiraceae*, *Bilophila*, *Dialister* and *Roseburia*. Maternal BMI, fat mass, triglyceride and insulin levels were positively associated with *Bilophila*. Correlations of bacterial abundance with diet intake showed that *Ruminococcus* and *Paraprevotella* were associated with total fat and unsaturated fatty acid intake, while *Collinsella* and *Anaerostipes* were associated with protein intake. While causal relationships remain unclear, collectively, these findings indicate pregnancy- and maternal obesity-dependent interactions between dietary factors and the maternal gut microbiome.

## 1. Introduction

Maternal obesity prior to pregnancy is a leading risk factor for greater life-time risk of obesity in children [1]. Findings from animal models unequivocally show that maternal obesity (OB) and/or obesogenic diets during pregnancy persistently augment offspring obesity risk. Women entering pregnancy with obesity are at greater risk for negative obstetric and perinatal outcomes, and there is greater likelihood of their offspring being large-at-birth and developing obesity later in life [2,3]. Multiple mechanisms have been hypothesized to contribute to the transmission of obesity to the child, including epigenetic changes and alterations in stem cells, in utero programming of nutrient utilization pathways, vertical transmission of the gut microbiome at birth and differences in human milk composition [4,5,6]. While robust associative evidence between maternal obesity, excessive gestational weight gain (GWG) and offspring obesity risk exists [7], recent evidence has suggested that the mother’s diet quality can also impact offspring adiposity [8]. However, clinical studies to support and understand the mechanisms via which maternal dietary composition impacts offspring obesity risk remain sparse.

Recent studies have demonstrated the importance of the gut microbiome in nutrient absorption, weight maintenance and the development of metabolic dysfunction [9,10]. More specifically, microbiota changes have been associated with obesity and excessive weight gain in adults [11,12]. The gut microbiome is continually shaped by a variety of factors, including genetics, xenobiotics, including medications, and host health [6,13]. Additionally, diet and dietary patterns have been shown to dominantly influence microbial diversity and affect host physiology [14]. During pregnancy, studies examining the gut microbiome have also shown changes in gut microbiome diversity and composition, although the results are conflicting [4,15,16,17]. Hence, studies are needed to clarify whether pregnancy alters the maternal gut microbiome and whether these changes are influenced by maternal health and dietary composition.

In the present study, we examined gut microbiome changes across pregnancy by examining fecal samples at 12, 24 and 36 weeks of gestation. We also investigated the effects of pre-pregnancy BMI by comparing women entering pregnancy at a normal weight (NW: BMI 18.5–24.9 kg/m^2^) compared to overweight/obese (OW/OB: BMI >25 kg/m^2^), and excessive GWG in pregnancy, controlling for maternal obesity status. Lastly, we assessed the influence of maternal diet on the microbiome during gestation. Our results suggest that microbial composition is altered by pregnancy status, pre-pregnancy BMI and intake of dietary fats.

## 2. Materials and Methods

### 2.1. Study Design, Participants and Data Collection 

**Figure 1:**
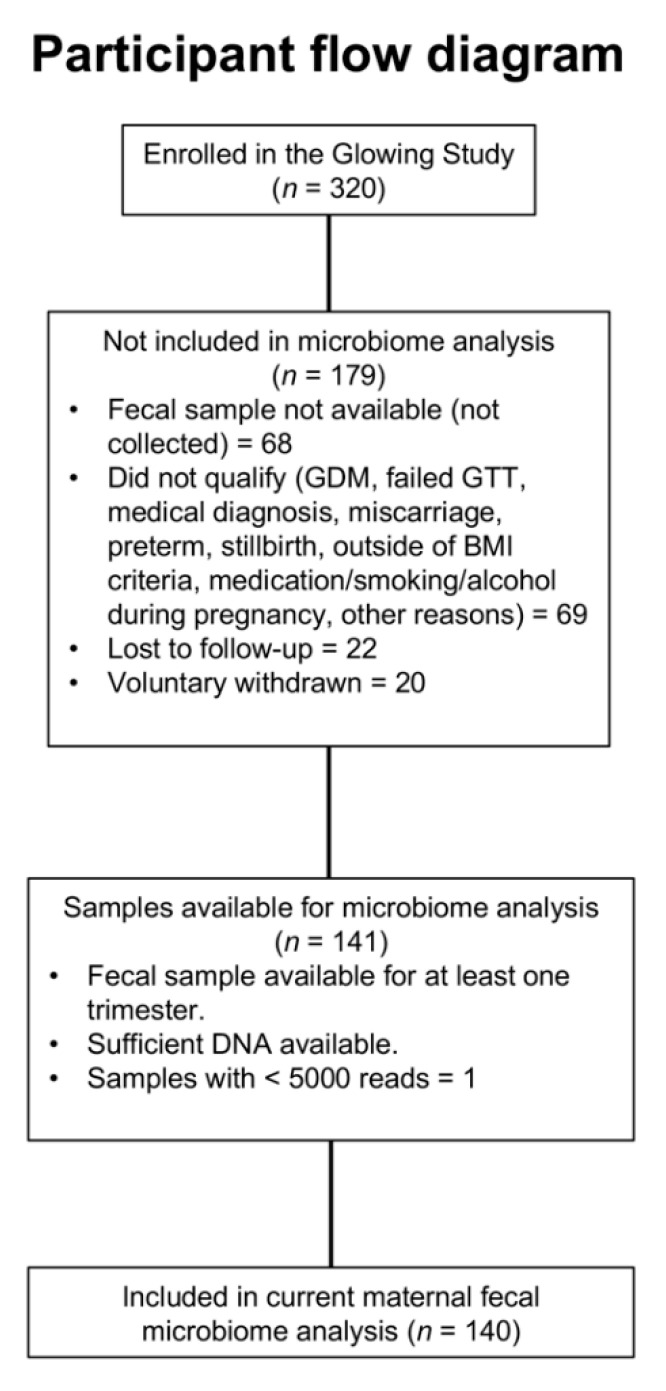
Participant flow diagram depicting subjects included in the current analyses.

Participants were enrolled as part of the Growing Life, Optimizing Wellness study (GLOWING, ClinicalTrials.gov ID: NCT01131117) at the Arkansas Children’s Nutrition Center. All participants were healthy, 2nd-parity women enrolled either pre-conception or at less than 10 weeks of gestation. Participants were recruited and grouped by pre-pregnancy body mass index (BMI) into either normal weight (NW; BMI 18.5–24.9) or overweight/obese (OW/OB; BMI 25–35). All procedures were approved by the Institutional Review Board at the University of Arkansas for Medical Sciences, and written informed consent was obtained prior to enrollment from all participants. Self-reported race, age, education, income, date of last menstrual period and estimated delivery date were obtained at enrollment. Exclusion criteria included preexisting medical conditions, use of fertility treatments, gestational diabetes, preeclampsia and other pregnancy complications. Data were collected at 12, 24 and 36 weeks of gestation for weight, metabolic/endocrine assessments, stool samples and dietary intake. Stool samples were collected for microbiome analyses as described below. We collected self-reported antibiotic and probiotic use from participants at each visit, documenting whether there was any use between their last visit and the current one (6-week duration between visits; duration of use was not documented). Approximately 12% of participants took antibiotics and 2% consumed probiotics over the duration of pregnancy. The participant flow diagram is shown in Figure 1.

### 2.2. Anthropometric and Body Composition Measurements

Body weight was measured at enrollment, 12 weeks and every 6 weeks thereafter. The difference between body weight at 12 and 36 weeks was used to compute gestational weight. Adherence to the Institute of Medicine (IOM) GWG guidelines was adjusted to reflect the last measure of weight at gestation week 36. All weights were measured to the nearest 0.1 kg on a tared scale (Perspective Enterprises, Portage, MI, USA) at the research facility. Standing height was also obtained using a standard wall-mounted stadiometer to the nearest 0.1 cm (Tanita Corp., Tokyo, Japan) at enrollment. Body composition measurements of fat mass (FM, kg) and fat free mass (FFM, kg) were assessed at enrollment using whole-body air displacement plethysmograph (BodPod, Cosmed, Concord, CA, USA) following standardized procedures. %FM was calculated by the following equation: (FM [kg]/body mass [kg] × 100%). Assays for circulating glucose, insulin, triglycerides (TG), non-esterified fatty acids (NEFA), HDL and LDL cholesterol at each trimester were performed in plasma using a clinical analyzer (Randox Laboratories, WV, USA).

### 2.3. Dietary Analysis

Dietary intakes were assessed using 3-day food records analyzed with the Nutrient Data System for Research software (NDSR; Nutrition Coordinating Center; University of Minnesota, MN, USA) at 12, 24 and 36 weeks of pregnancy. Daily dietary nutrient intakes were averaged over the 3 days, which included a weekend day for each time point. For simplicity, overall dietary composition over pregnancy was derived by averaging dietary data from all three data points (12, 24 and 36 week) for each participant.

### 2.4. Microbiome Analysis via 16S rRNA Amplicon Sequencing

Stool samples were self-collected by participants either at the research visit or within 7 days prior to their 12-, 24- and 36-week visits in a sterile cup. Participants were instructed to freeze samples immediately after collection and to transport the sample on ice to the research center. Samples were later stored in the research laboratory at −70 °C until processing. Bacterial DNA from ~100 mg of stool was isolated using the QIAamp Fast DNA stool mini kit (Qiagen; Hilden, Germany), including a bead-beating step (Precellys homogenizer). Fifty nanograms of genomic DNA was used for amplification of the V4 variable region of the 16S rRNA gene using 515F/806R primers. Dual-indexed forward and reverse primers were used as described by Kozich et al. [18] to accommodate multiplexing of up to 384 samples per run. Paired-end sequencing (2X 250 bp) of pooled amplicons was performed on a MiSeq Instrument (Illumina; San Diego, CA, USA) using ~30% PhiX DNA.

### 2.5. Microbial Ecology Data Analysis

We utilized QIIME (v1.9.1) and in-house scripts to process and filter high-quality reads following previously established methods [19]. PEAR was used to stitch paired reads and Phred quality scores were used to further filter assembled reads [20]. USEARCH61 was used to remove chimeric reads [21]. Filtered reads (mean counts per sample = 37,971) were demultiplexed within QIIME, and samples with less than 5000 reads were excluded from further analysis. We clustered sequences into operational taxonomical units (OTUs, based on >97% identity) with UCLUST. OTU picking was performed using the open-reference method, which involved clustering reads against a reference sequence collection and performing de novo OTU picking on the reads that failed to align to any known reference sequence in the database [22]. Resulting OTU tables were checked for mislabeled sequences to eliminate any erroneous mislabeling [23]. Representative sequences were further aligned using PyNAST with the Greengenes core-set alignment template [24]. The default (FASTTREE) method in QIIME was performed for the construction of the phylogenetic tree [25]. Ecological diversity measures and identification of group differences in bacterial taxonomic abundance are described below.

All statistical analyses and visualizations were performed in R (version 4.05). The microbiota counts, sample metadata and taxonomy information were imported using the *phyloseq* package [26]. Preprocessing included retaining taxa with 5 counts in a minimum of 5% of samples (using the core function in the *microbiome* package), agglomerating at each taxonomic rank and generating taxonomic abundance. Stacked bar plots (for individual samples and group means), iris plots and heatmaps of most abundant taxa were generated using the vegan, *microeco* and *micoviz* packages [27,28,29]. Derivations of alpha-diversity indices (Chao1, Shannon, Simpson and Fisher indices) at the genus level were done using vegan and *microeco* packages. Group differences in α-diversity measures were assessed by ANOVA or t-test using the *compareGroups* package, including pair-wise comparisons in the case of more than two groups [30]. Repeated-measures ANOVA was used to assess longitudinal changes across pregnancy for subjects with samples in all trimesters. Unsupervised principal components analysis (PCA) of genus-level taxa and biplot representation was done using the *microviz* package following centered-log ratio transformation [29]. Between-sample diversity (β-diversity) was assessed using Jaccard similarity or Aitchison distance and visualized using non-metric multi-dimensional scaling (NMDS) ordination. Group differences in β-diversity were assessed on the dissimilarity matrix using permutational multivariate analysis of the variance (PERMANOVA) with 999 permutations and via comparing the distance between groups using the Wilcox test or ANOVA.

Associations of genus-level taxa abundance and circulating metabolic markers (glucose, insulin, HDL, LDL cholesterol, triglycerides, NEFA) and maternal early pregnancy BMI and fat mass (measured via BODPOD) were assessed using the *microeco* package [28]. Distance-based redundancy analysis (db-RDA) Bray–Cutis distances were used to summarize relationships between microbial taxa abundance and metabolic traits. db-RDA is a method for carrying out constrained ordinations on data using non-Euclidean distance measures. For correlation clustering of genera with metabolic variables, we first extracted a set of differential features using random forest, which were used for correlation using Spearman correlation. Correlation-based networks were constructed using the trans_network function in the *microeco* package [27]. Network attributes including nodes, modules and eigenvalues were calculated using the cal_module and cal_eigen functions. Associations of modules with metabolic variables were then performed using Spearman correlations with the cal_cor function in *microeco*. Functional analysis of genus-level microbial taxa was performed using the FAPROTAX database [31]. Correlations of functional pathways with metabolic traits were performed using Spearman correlation and visualized as heatmaps. Multivariable associations between groups and taxonomic abundance were assessed using the *MaAsLin2* package [32], adjusting for covariates as necessary. The main group effects (maternal obesity status, IOM weight gain category or trimester of pregnancy) were considered fixed effects, whereas participant identifier, stage of pregnancy and obesity status were included as random effects in the multivariable models, depending on the main effects examined. Before analysis with *MaAsLin2*, taxa were agglomerated to either family or genus levels and considered separately. The linear model (lm) function was utilized on total sum scaled (TSS) normalized data with minimum prevalence (5%) and standardization of covariates to z scores. For analysis of changes over pregnancy, samples at 12 week (trimester 1, T1) were assigned as reference. All *p*-values were false discovery rate-adjusted (Benjamini–Hochberg, q-values) and features with q < 0.25 were considered significant. The OTU relative abundance is presented as the percent relative abundance when described in the text. Associations between genus-level taxonomic abundance and selected dietary variables were assessed using Spearman’s correlations using the *corrplot* package. Correlations were considered significant at nominal *p* < 0.05 and not corrected for multiple testing due to the exploratory nature of the analyses.

## 3. Results

### 3.1. Participant Characteristics

Overall, the analyses included 140 pregnant women grouped by early pregnancy BMI into either NW (*n* = 60) or OW/OB (*n* = 80) groups (Table 1). The average age of participants was 29 years and 87% identified as Caucasian. As anticipated by the study design, there were significant differences in BMI and fat mass between OW/OB and NW women. In this cohort, most women in the OW/OB category were overweight (74%; average BMI = 27.41 kg/m^2^) and only 26% were women with obesity (average BMI = 32.5 kg/m^2^). More women in the OW/OB group had GWG above IOM guidelines (58%) compared to in the NW group (22%) (Table 1).

### 3.2. Alpha-Diversity across Pregnancy

To examine changes in the maternal gut microbiome across pregnancy, we assessed data at 12 weeks (T1), 24 weeks (T2) and 36 weeks (T3) of gestation. We calculated Chao1, Shannon, Simpson and Fisher indices of alpha-diversity. There were no significant effects across trimester for any measures of α-diversity (*p* > 0.05, Table 2). When restricted to subjects with longitudinal data available at all three trimesters (*n* = 56), there was a significant effect of pregnancy on evenness (Simpson index; *p* = 0.019), with a decrease between T1 and T2 using repeated-measures ANOVA. Other measures of α-diversity were not found to be different.

### 3.3. Microbiota Composition and Abundance across Pregnancy

**Figure 2:**
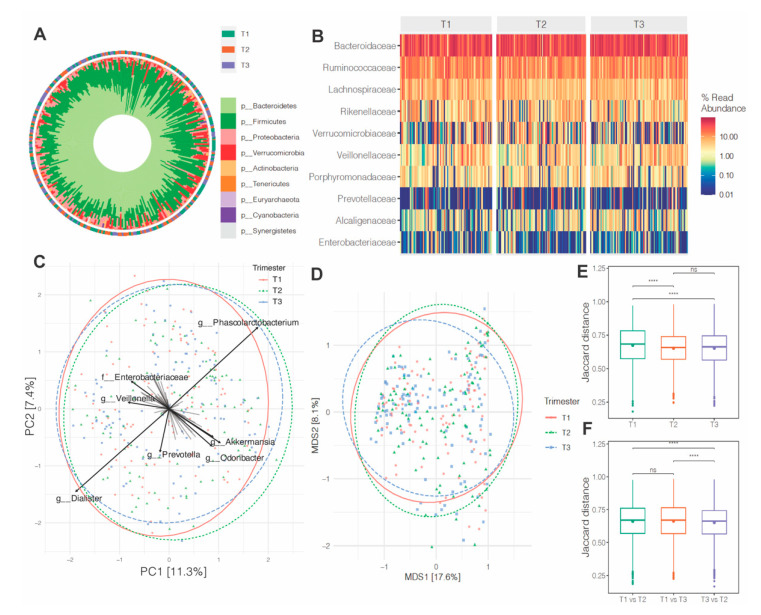
Maternal gut microbiome composition over pregnancy. (**A**,**B**) Phylum- and family-level abundances of bacteria at trimesters 1, 2 and 3 of pregnancy. Sample sizes across trimesters: T1 (*n* = 101), T2 (*n* = 98) and T3 (*n* = 107). Microbial taxa levels were assessed via sequencing of 16S rRNA gene amplicons (V4 region). (**C**) Bi-plot representation of principal components analysis of genus-level taxa. (**D**) Non-metric dimensional scaling (NMDS) ordination of Jaccard similarities of samples from all three trimesters. (**E**) Boxplots of group and (**F**) intergroup Jaccard distances. Group differences were determined by one-way ANOVA across the three trimesters and via PERMANOVA (999 permutations) for beta-diversity. **** indicates *p* < 0.01.

The distribution of bacterial taxa at the phylum level at each trimester (Figure 2A) showed that the maternal gut microbiome is dominated by Bacteroidetes and Firmicutes. At the family level, *Bacteroidaceae*, *Ruminococcaceae* and *Lachnospiraceae* showed the highest abundance, with broadly conserved patterns across pregnancy (Figure 2B). Unsupervised PCA analysis and NMDS ordination of Jaccard similarity showed no significant differences in global genus-level composition over pregnancy. These were assessed via comparisons of the main principal components across groups (for PCA) and PERMAOVA for Jaccard similarities (Figure 2C,D). However, pairwise comparisons of each trimester showed significant differences between trimester 1 and others (2 and 3), but not between trimesters 2 and 3 (Figure 2E,F). We also analyzed β-diversity comparing antibiotic and probiotic use versus no use during pregnancy and found no significant differences (data not shown). Exclusion of participants who used antibiotics or probiotics did not substantially alter the results; therefore, we included these participants in all analyses.

**Figure 3:**
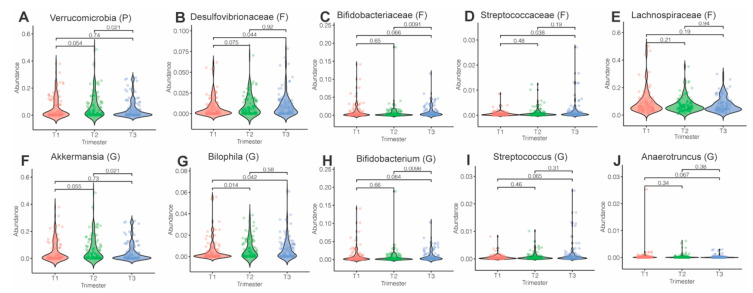
Violin plots showing levels of differentially expressed taxa over pregnancy. (**A**) Phylum Verrucomicrobia, (**B**) family *Desulfovibrionaceae*, (**C**) family *Bifidobateriaceae*, (**D**) *family Streptococcaceae*, (**E**) family *Lachnospiraceae* and (**F**–**J**) genera *Akkermansia*, *Bilophila*, *Bifidobacterium*, *Streptococcus* and *Anaerotruncus*. Differential abundance was assessed using *MaAsLin2*. Sample sizes across trimesters: T1 (*n* = 101), T2 (*n* = 98) and T3 (*n* = 107). All main effects of pregnancy were *p* < 0.05 (q < 0.2). Pairwise *p*-values were derived using Wilcoxon test.

We further employed *MaAsLin2* to examine changes in specific taxa over pregnancy. At the phylum, family and genus levels, evaluating the effects of pregnancy stage showed significant changes in phylum Verrucomicrobia, with a marked decrease in trimester 3 (Figure 3A). Levels of *Desulfovibronaceae*, *Bifidobacteraceae* and *Streptococcaceae* increased over pregnancy, while *Lachnospiraceae* family abundance decreased over pregnancy. Consistent with these findings, levels of genus *Akkermansia* decreased between trimesters 2 and 3, while levels of *Bilophila*, Bifidobacterium and Streptococcus increased over the course of pregnancy (Figure 3B–J). Analysis of only subjects with longitudinal data further revealed a change across trimesters in the abundance of phyla Actinobacteria and Verrucomicrobia and genus *Lachnospira* (*p* < 0.05, data not shown).

### 3.4. Effects of Maternal Obesity on Microbiota Composition and Abundance

To evaluate the effects of maternal OW/OB status on α-diversity, we examined measures of richness and evenness in all pregnancy stages combined and at each trimester. Maternal obesity status was not associated with changes in α-diversity measures between groups (Table 3).

**Figure 4:**
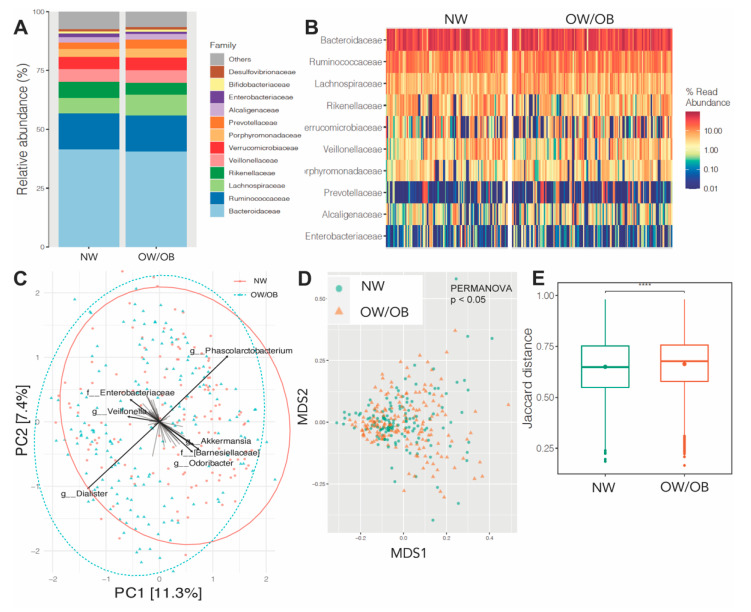
Maternal obesity status and gut microbiome composition. (**A**,**B**) Family-level composition of gut microbiome in women with either normal weight (NW) or overweight/obese (OW/OB) BMI. Group differences were assessed via Student’s t-test. (**C**) Bi-plot representation of principal components analysis of genus-level taxa. (**D**) Non-metric dimensional scaling (NMDS) ordination of Jaccard similarities of samples from both NW and OW/OB groups. (**E**) Boxplots of Jaccard distances by group. Group differences were determined by Wilcoxon tests or via PERMANOVA (999 permutations) for beta-diversity. **** indicates *p* < 0.01.

Taxonomic abundance analyses showed that the top five families, *Bacteroidaceae*, *Ruminococcaceae*, *Lachnospiraceae*, *Rikenellaceae* and *Veillonellaceae*, accounted for 75% of all bacteria in both groups (Figure 4A,B). Unsupervised PCA and NMDS ordination of Jaccard similarity values (β-diversity) showed significant differences in global genus-level composition between NW and OW/OB groups (PERMANOVA *p* < 0.05) (Figure 4C,D). Boxplots of Jaccard distances between NW and OW/OB groups also showed significant differences (Figure 4E).

**Figure 5:**
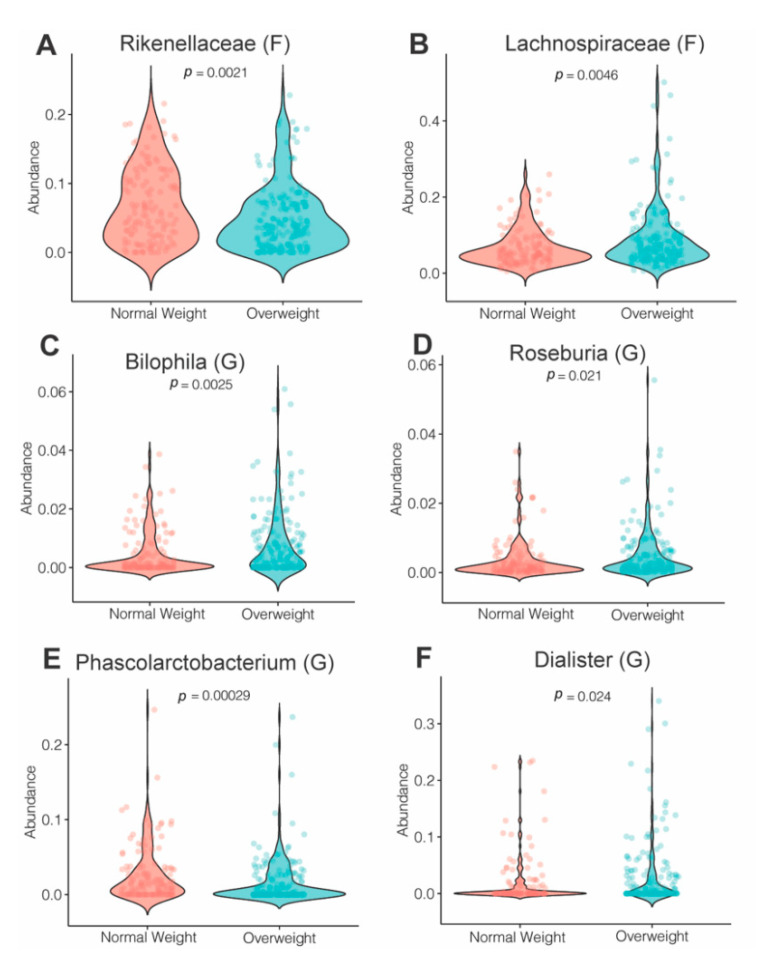
Maternal microbiome changes associated with obesity status. (**A**) Violin plots showing levels of differentially expressed bacteria associated with maternal OW/OB status. (**A**) Family *Rickenellaceae*, (**B**) family *Lachnospiraceae* and (**C**–**F**) genera *Bilophila, Roseburia*, *Phascolarctobacterium* and Dialester. Statistical differences between the groups were determined by *MaAsLin2*, adjusting for pregnancy trimester. All of the tests were corrected for multiple comparisons using the false discovery rate (FDR) (q < 0.2). (*n* = 132 and 174 in NW and OW/OB groups).

We next assessed the effects of maternal OW/OB status on family- and genus-level bacterial abundance. At the family level, the abundance of *Rikenellaceae* was decreased in the OW/OB group, while levels of *Lachnospiraceae* were increased in subjects that were OW/OB compared to NW (*p* < 0.005) (Figure 5A,B). Genus-level analysis showed that maternal overweight was associated with increased levels of *Biolphila*, *Roseburia* and *Dialister* and decreased levels of *Phascolarctobacterium* (Figure 5C–F).

**Figure 6:**
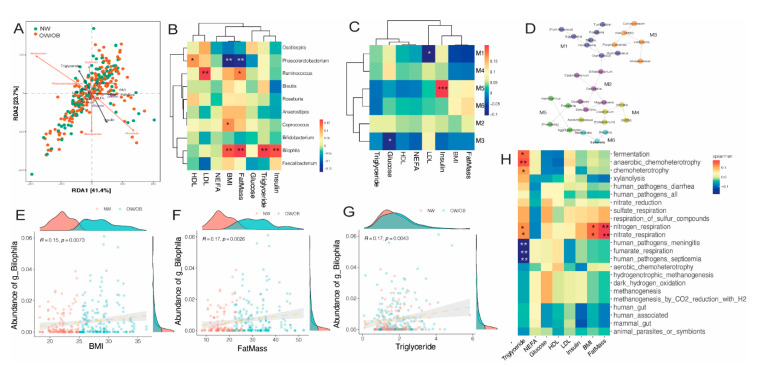
Associations between maternal microbiome and metabolic and anthropometric markers. (**A**) Distance-based redundancy analysis (db-RDA) of bacterial abundance and associations with metabolic and anthropometric markers. Bray–Curtis distances were measured using genus-level abundance. Maternal BMI and fat mass were measured in early pregnancy (< 12 weeks). Serum parameters were assessed at each trimester. (**B**) Hierarchical clustering of correlations between top 10 differential genera and metabolic markers. For correlation heatmaps, red to blue spectrum denotes positive to negative associations, respectively. (**C**,**D**) Correlation-based network and associations between network modules and metabolic markers. Scatterplots showing abundance of genus *Bilophila* and (**E**) maternal BMI, (**F**) maternal fat mass and (**G**) triglyceride levels. (**H**) Correlation between inferred bacterial function abundance and metabolic markers. ** indicates *p* < 0.001; * indicates *p* < 0.05.

Distance-based redundancy analysis of genus-level taxonomic abundance and metabolic and adiposity variables (maternal BMI, fat mass, serum glucose, insulin, TG, HDL and LDL cholesterol) showed the alignment of maternal BMI and fat mass with the *Dialister* genus, and *Akkermansia* and *Blautia* abundance with serum glucose levels (Figure 6A). Correlation analysis with metabolic variables further indicated a positive correlation between maternal fat mass, BMI, insulin and TG levels with genus *Bilophila* and a positive association of genus *Ruminococcus* with serum LDL levels. *Phascolarctobacterium* levels were negatively associated with maternal BMI and fat mass (Figure 6B). Network analysis of bacterial taxa showed six associated modules (Figure 6D). Correlation of modules with metabolic variables showed a strong positive association between module 5 (enriched for *Prevotella*, *Aggregatibacter* and *Hemophilus*) and serum insulin values (Figure 6C). Associations between *Bilophila* abundance and early pregnancy maternal BMI, fat mass and serum triglycerides are presented in Figure 6E,F. Predicted microbial functions were also correlated with maternal metabolic variables (Figure 6H). These analyses showed a positive association between nitrogen/nitrate respiration and maternal BMI and fat mass. Serum triglyceride levels were negatively associated with fumarate respiration and positively associated with anaerobic chemoheterotrophy. A high-resolution version of this figure is also included in the Supplementary Materials (Figure S1).

### 3.5. Impact of Diet on Maternal Gut Microbiome in Pregnancy

**Figure 7:**
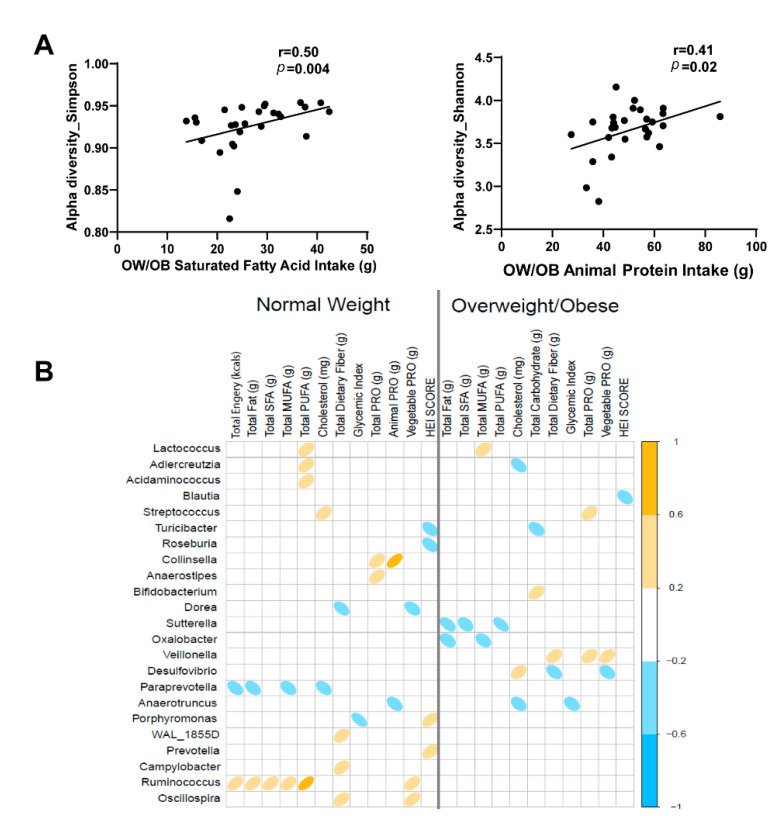
Associations between maternal diet and microbiome in pregnancy. (**A**) Associations between α-diversity indices and saturated fat intake and animal protein intake in pregnant women. Dietary intake and genus-level abundance were averaged across all trimesters from women who had both datasets in all three trimesters (NW: *n* = 25, OW/OB: *n* = 31). Nominal *p*-values without multiple testing correction are shown. (**B**) Correlation matrix showing significant associations (*p* < 0.05) between dietary intake variables and genus-level abundance. Yellow and blue denote positive and negative associations, respectively.

To better understand the relationship between diet and the gut microbiome in pregnancy, we examined associations with α-diversity and genus-level abundance. Diet records were analyzed using NDSR software and averaged over pregnancy for participants with complete data. Genus-level abundances were also averaged over pregnancy in participants who provided stool samples during all three trimesters. Examination of dietary intake variables between NW and OW/OB groups showed no major differences. Spearman correlation of α-diversity measures and dietary intake of specific nutrients revealed that saturated fatty acid (SFA) intake was positively correlated with the Simpson index in OW/OB subjects (r = 0.50, *p* = 0.004, Figure 7A). Likewise, within OW/OB participants, animal protein intake was positively correlated with the Shannon index (r = 0.41, *p* = 0.02, Figure 7A). Finally, we observed several significant associations between genus-level abundance and dietary intake in both NW and OW/OB women (Figure 7B). Notably, in NW subjects, *Ruminococcus* showed significant positive associations with energy, fat, SFA, monounsaturated fatty acid (MUFA) and polyunsaturated fatty acid (PUFA) intake. In addition, the abundance of *Paraprevotella* was negatively associated with total energy, total fat and MUFA intake. *Collinsella* was positively associated with both total protein and animal protein intake, and *Anaerostipes* was positively associated with total protein intake. In the OW/OB group, *Sutterella* was negatively associated with total fat, SFA and PUFA intake, and the abundance of *Veillonella* was positively associated with dietary fiber, total protein and vegetable protein intake.

### 3.6. Effects of Gestational Weight Gain on Microbiota Composition and Abundance

**Figure 8:**
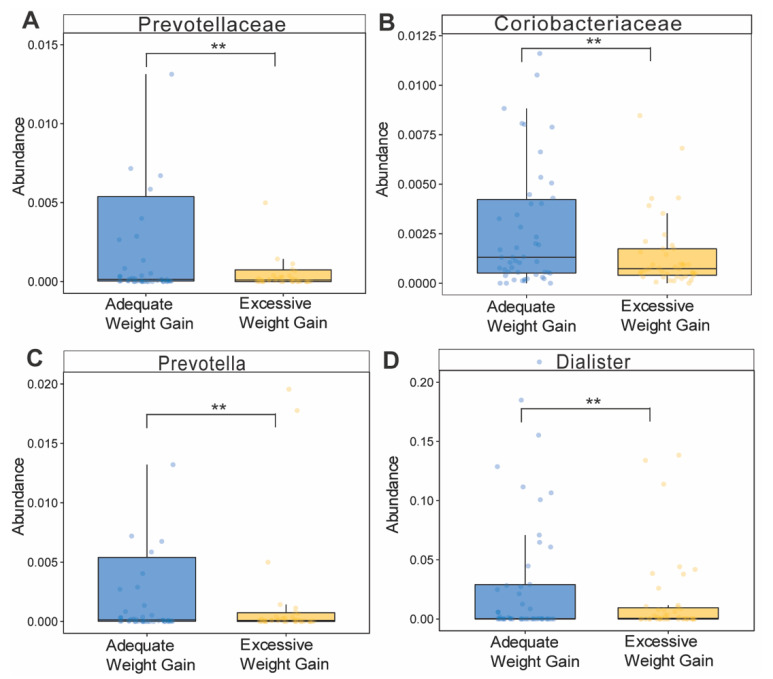
Gestational weight gain effects on bacterial abundance. (**A**–**D**) Genus- and family-level abundance of bacteria at trimester 3 (T3) of women with either adequate or excessive weight gain. Statistical differences between the groups were assessed using linear modeling, adjusting for early pregnancy BMI. Significance was set at *p* < 0.05, using nominal *p*-values. No bacterial taxa showed significance following multiple testing correction. Sample sizes included for analyses (adequate weight gain: *n* = 50, excessive GWG: *n* = 42). ** indicates *p* < 0.01.

Since excessive gestational weight gain (exGWG) is closely associated with detrimental outcomes for mother and child, we examined how exGWG altered gut microbiota composition and/or abundance when controlling for BMI. We classified GWG in women based on the IOM guidelines [33]. For this analysis, we investigated whether exGWG compared to adequate weight gain, independent of BMI, altered the gut microbiome. We found no differences in α- or β-diversity when comparing adequate versus excessive GWG (data not shown). However, at the family and genus level, exGWG was associated with decreased abundance of *Prevotella* (genus), *Dialister* (genus), Prevotellaceae (family) and Coriobacteriaceae (family) at trimester 3 (T3, nominal *p* < 0.05, Figure 8).

### 3.7. Discussion

A multitude of factors influence the development of obesity and related co-morbidities. Of these, maternal diet and body composition are important determinants of the risk of obesity in childhood and later in life. Among the hypothesized mechanisms involved in the intergenerational transmission of obesity risk is a persistent change in the offspring microbiome. Nonetheless, the impact of obesity on the maternal gut microbiome in human studies is not exhaustively known. Several novel findings are evident from the present study: (1) pregnancy stage was associated with clear alterations in specific gut microbial taxa and changes in diversity, especially between the first and third trimester; (2) pre-pregnancy obesity has a robust effect on microbial diversity and the levels of important taxa; and (3) maternal diet composition and metabolic biomarkers are associated with altered α-diversity and bacterial abundances. These findings represent a comprehensive analysis of the associations between maternal diet and microbial patterns in pregnancy.

An important aspect of the present experimental design is the collection of samples during all three trimesters, permitting temporal analysis. Previous work focused on either early/late pregnancy, delivery or post-partum [15,16,34]. Due to variations in sample collection, timing and methods of microbial analyses, prior data are conflicting on whether pregnancy alters the diversity measures of the microbiome. Our findings indicate that while pregnancy stage did not substantially influence α-diversity, β-diversity measures were altered in the latter part of pregnancy. Our study only included mothers of second parity. The rationale for including only second-parity mothers was based on limiting variation in neonatal parameters associated with parity and to provide greater homogeneity among participants. Additionally, specific microbial abundances were altered across trimesters. Specifically, we identified decreases in *Akkermansia* and *Lachinospiraceae*, and increased Actinobacteria and *Streptococcus* abundance across pregnancy. These findings are consistent with previous reports of microbiome changes during pregnancy [15,35]. A recent study of pregnant women in Southern China analyzed the gut microbiome profiles of 1479 women along with 132 host-related meta-variables [17]. Consistent with our findings, this study found a greater effect size of maternal pre-pregnancy weight/BMI relative to gestational stage on the microbiome. Moreover, the findings from this cohort also indicated no changes with α-diversity measures over pregnancy, but significant alterations in specific taxa. Consistent with our observations, the abundances of *Lachnospiraceae*, *Akkermansia* and *Streptococcus* were decreased over gestation. Given the differences in sample size, the previous study was significantly more powered to detect smaller changes. The functional relevance of these specific alterations during pregnancy remains unclear. However, a number of studies have indicated links between lipid metabolism, insulin sensitivity and gut health markers and the abundance of *Akkermansia* and *Lachnospiraceae* family members. Since pregnancy is associated with robust changes in insulin sensitivity and lipid metabolism, including serum triglyceride levels, interactions between these processes are a possibility. Along these lines, the transfer of microbiota from late pregnancy into germ-free mice does diminish insulin sensitivity [15].

Obesity status was associated with robust changes in the microbiome during pregnancy. Similar to our findings, prior studies showed decreases in *Lachinospiraceae* in the context of obesity, diabetes or excess gestational weight gain [4,36]. Clinical studies examining the effects of pregnancy on Verrucomicrobia are limited, although it was previously shown to be decreased in obese individuals [37]. The gut microbiome plays a key role in body weight and metabolic regulation by impacting nutrient absorption, energy balance and appetite [34]. Mouse models of obesity (using both genetic and diet-induced obesity) show robust and reproducible changes in the gut microbiome. However, data from human studies are far more equivocal. Indeed, previous studies have shown that the large interindividual variation present in the human microbiome contributes to the much more modest differences observed in human obesity [38,39]. Recent studies suggest that maternal body weight prior to pregnancy has a measurable impact on the microbiome [17]. In our study, maternal weight status was associated with changes in β-diversity in pregnancy. In line with previous studies, maternal OW/OB status was associated with greater *Lachnospiraceae*, including genus *Roseburia* [17]. Likewise, in a study of 1914 Chinese adults, *Roseburia* among other genera was discriminative of obesity status [40]. In another study of weight loss in predominantly female subjects, greater *Dialister* and lower *Phascolarctobacterium* levels were associated with a lack of weight loss in individuals with obesity [41]. Abundance of *Phascolarctobacterium* and *Dialister* has been associated with insulin sensitivity (positively and negatively, respectively) in individuals with obesity, as measured by the hyperinsulinemic–euglycemic clamp technique [42]. Another notable alteration associated with maternal obesity status was increased levels of the genus *Bilophila*. *Bilophila* levels which were also strongly associated with maternal BMI, fat mass, insulin and triglyceride levels. The genus *Bilophila* contains one known species, *B. wadsworthia*, which is an anaerobic, asacchrolytic, sulfite-reducing, bile resistant bacillus [43]. *Bilophila* is considered to be a pathobiont that expands in the presence of dietary lipids and aggravates metabolic syndrome and intestinal inflammation in mice [44,45]. Diets high in fats reproducibly increase the abundance of *Bilophila* and are associated with increased bile acids, as does intake of taurocholic acid [46,47]. Short-term feeding studies in humans also indicate that animal-based diets increase the abundance of *Bilophila* and other bile-resistant bacteria [48]. In mice, taurine conjugation of hepatic bile acids increases the availability of organic sulfur, which promotes the abundance of *B. wadsworthia*. A recent report also showed that in women who developed intrahepatic cholestasis during pregnancy (ICP), greater total bile acids was associated with greater *Bilophila* in the gut microbiome [49]. Overall, maternal obesity may be associated with a combination of dietary fat intake and dysregulation of bile acid metabolism, which may contribute to the enrichment of *Bilophila*.

A salient feature of the present findings is the careful consideration of maternal diet during pregnancy. Despite the wide recognition of the predominant influence of diet on the gut microbiome, no studies to date have longitudinally assessed maternal dietary patterns along with the gut microbiome during pregnancy. Identifying dietary components that associate with the gut microbiome in pregnancy is critical, as a number of dietary intervention strategies to mitigate weight gain and improve metabolic health in pregnant women have been proposed. Two noteworthy observations are evident from the present work. First, maternal obesity modifies the associations between dietary components and the microbiome. Second, maternal obesity and diet influence distinct subsets of microbial taxa. One notable change in women with NW was with the abundance of *Ruminococci*, which were positively associated with fat intake, with PUFA intake showing the strongest correlation. Similarly, previous studies have shown increased abundance of *Ruminococcus* with omega-3 (PUFA) supplementation as well as with animal protein intake [48,50]. Pregnant women who had diets high in plant-rich foods and fatty acids, specifically PUFA, had a higher abundance of *Ruminococcus* [51]. The results from our study suggest that PUFA intake may contribute to greater *Ruminococcus* abundance in women with normal weight. We also identified *Collinsella* to be positively associated with total protein and animal protein intake in women with NW. *Collinsella* has previously been shown to have a negative correlation with dietary fiber [14] and a positive correlation with dietary fat [52]. Although there are limited data suggesting a relationship between dietary protein intake and *Collinsella*, diets high in animal protein typically have higher fat intake. Finally, in NW subjects, we observed negative associations between *Paraprevotella* and total fat, MUFA and cholesterol intake, which has been previously shown to be negatively associated with serum triglycerides and LDL cholesterol [53]. We also noted associations between microbial abundance and dietary intake in women with OW/OB BMI that were not present in NW women. *Sutterella*, which has been found to be negatively associated with obesity in adults and pregnant women [54,55], was negatively associated with total fat, saturated fat and PUFA intake in women with OW/OB. *Veillonella* was positively associated with dietary fiber, total protein and vegetable/plant-based protein. The underlying drivers of these associations specifically in overweight individuals is not clear. However, differences in dietary host metabolism, dietary patterns and the levels of processed foods may contribute to these differences.

The present study is not without limitations. Women in this study were generally healthy, with the majority of women having a BMI in the overweight rather than obese category. Therefore, greater differences within the gut microbiome may be more likely to be observed in women who have more severe obesity and metabolic dysregulation. Women in the current report were also devoid of gestational diabetes, which may be associated with more robust changes in gut microbiota. Our diet analysis averaged individual diets over all three trimesters of pregnancy due to the small sample sizes. This may have diminished changes in the dietary intake of specific nutrients over pregnancy. Our data still provide evidence that dietary intake during pregnancy is associated with gut microbiome profiles and should be studied more extensively. Many differences in the present report were only nominally significant and did not pass multiple testing corrections, and hence should be interpreted with caution. Lastly, since 16S rRNA amplicon sequencing does not allow for species-level resolution, the present analyses reflect a broad view of the microbiome and leave further room for more granular analyses.

In conclusion, our findings indicate pregnancy- and maternal obesity-dependent interactions and dietary factors in the maternal microbiome during gestation. Dietary intake during pregnancy is associated with α-diversity indices and genus abundance levels. More specifically, our findings point to a robust influence of maternal weight status, dietary fats, fatty acids and protein type on the abundance of bacteria associated with gut inflammation, obesity and metabolic function. These findings are consistent with the growing consensus that dietary interventions during pregnancy could in part mediate their beneficial effects by modulating the maternal gut microbiome and potentially impact offspring health.

## Figures and Tables

**Table 1:** Cohort characteristics.

	All	Normal Weight (BMI <25 kg/m^2^)	Overweight/Obese (BMI ≥ 25 kg/m^2^)	*p*-value (NW vs. OW/OB)
**N (%)**	140	60 (42.9)	80 (57.1)	
**Age at enrollment (years)**	29.46 ± 0.32	29.45 ± 0.51	29.47 ± 0.41	0.970
**Race**				
**Caucasian**	122 (87.1)	52 (86.7)	70 (87.5)	
**Non-Caucasian**	18 (12.9)	8 (13.3)	10 (12.5)	
**Body Mass Index (kg/m^2^)**	25.91 ± 0.34	22.14 ± 0.22	28.73 ± 0.31	<0.0001
**Fat Mass (%)**	25.47 ± 0.79	17.32 ± 0.57	31.58 ± 0.80	<0.0001
**Gestational Weight Gain (kg)**	12.01 ± 0.38	12.81 ± 0.38	11.41 ± 0.58	0.047
**IOM Weight Gain Category**				<0.0001
**Inadequate**	19 (13.7)	10 (16.9)	9 (11.3)	
**Adequate**	61 (43.9)	36 (61.0)	25 (31.3)	
**Excessive**	59 (42.4)	13 (22.0)	46 (57.5)	

Data presented as counts (%) or mean ± SEM. Group differences were determined by t-test for continuous variables with a significance level of *p* < 0.05.

**Table 2:** Alpha-diversity indices assessed over pregnancy trimesters.

Pregnancy Stage	T1	T2	T3	*p*-Value Overall	*p*.T1 vs. T2	*p*.T1 vs. T3	*p*.T2 vs. T3
***n***	*n* = 101	*n* = 98	*n* = 107				
**Chao1**	40.1 (5.46)	40.9 (5.92)	40.4 (5.55)	0.641	0.615	0.915	0.844
**Shannon**	2.02 (0.35)	2.00 (0.37)	1.96 (0.39)	0.61	0.938	0.59	0.805
**Inverse Simpson**	4.64 (1.96)	4.55 (1.87)	4.34 (1.78)	0.498	0.929	0.481	0.718
**Fisher**	4.38 (0.66)	4.43 (0.71)	4.37 (0.64)	0.767	0.829	0.996	0.777

Values are means (SD) for each measure of alpha-diversity calculated at the genus level. Overall *p*-values calculated by one-way ANOVA.

**Table 3:** Alpha-diversity indices associated with maternal obesity status.

	NW	Overweight/Obese	*p*-Value
**Chao1**	40.7 (5.23)	40.5 (5.77)	0.81
**Shannon**	2.00 (0.34)	1.99 (0.38)	0.766
**Inverse Simpson**	4.46 (1.82)	4.58 (1.95)	0.576
**Fisher**	4.38 (0.58)	4.38 (0.71)	0.949

Values are means (SD) for each measure of alpha-diversity calculated at the genus level. Overall *p*-values calculated by Wilcoxon test.

## Data Availability

Access to sequencing data is available on request. These have currently not been submitted to a public repository due to the ongoing nature of the studies.

## Supplementary Materials

The following are available online at https://www.mdpi.com/article/10.3390/nu13093295/s1, Figure S1: high-resolution version of Figure 6.
