# Supplementary figures and images for: Associations between Maternal Diet, Body Composition and Gut Microbial Ecology in Pregnancy
Source: Nutrients. 2021 Sep 21;13(9):3295. doi: 10.3390/nu13093295 (PMC8468685; doi:10.3390/nu13093295)

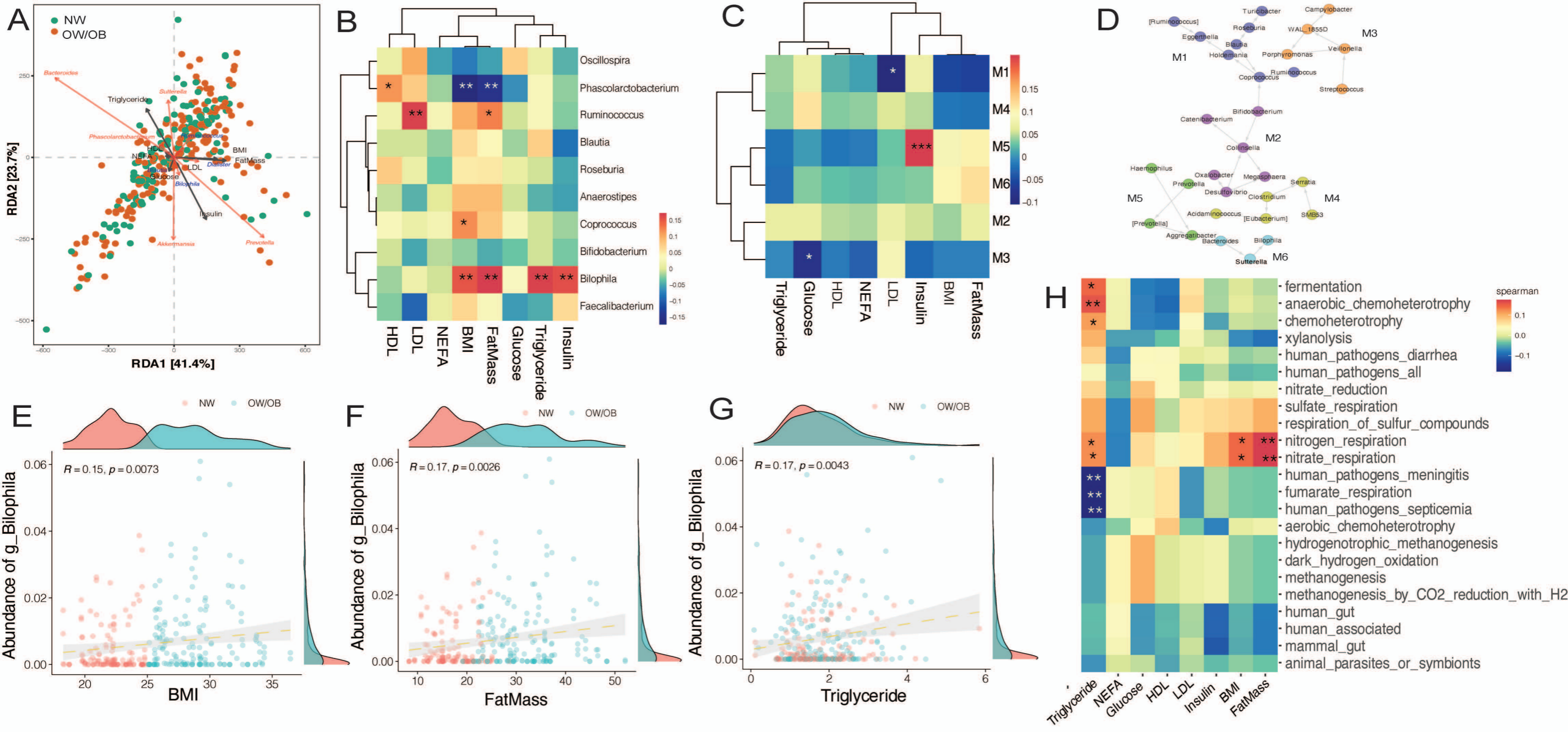

Supplement: Supplementary file 1 [file nutrients-13-03295-s001.zip › nutrients-1377635-supplementary.pdf]
